# Commercially Available Heart Rate Monitor Repurposed for Automatic Arrhythmia Detection with Snapshot Electrocardiographic Capability: A Pilot Validation

**DOI:** 10.3390/diagnostics12030712

**Published:** 2022-03-15

**Authors:** Chiara Martini, Bernardo Di Maria, Claudio Reverberi, Domenico Tuttolomondo, Nicola Gaibazzi

**Affiliations:** 1 Department of Radiology, Parma University Hospital, Via Gramsci 14, 43125 Parma, Italy; 2 Independent Researcher, 43126 Parma, Italy; dimaria.bernardo@gmail.com; 3 Poliambulatorio Città di Collecchio, Str. Nazionale Est, 4/A, 43044 Collecchio, Italy; claudioerre110153@gmail.com; 4 Non-invasive Cardiology, Parma University Hospital, Via Gramsci 14, 43125 Parma, Italy; d.tuttolomondo@hotmail.it (D.T.); ngaibazzi@gmail.com (N.G.)

**Keywords:** cardiovascular prevention, heart failure, sports cardiology, digital cardiology, arrhythmia, cardiac monitoring, sensors, electrocardiogram

## Abstract

The usefulness of opportunistic arrhythmia screening strategies, using an electrocardiogram (ECG) or other methods for random “snapshot” assessments is limited by the unexpected and occasional nature of arrhythmias, leading to a high rate of missed diagnosis. We have previously validated a cardiac monitoring system for AF detection pairing simple consumer-grade Bluetooth low-energy (BLE) heart rate (HR) sensors with a smartphone application (RITMIA™, Heart Sentinel srl, Italy). In the current study, we test a significant upgrade to the above-mentioned system, thanks to the technical capability of new HR sensors to run algorithms on the sensor itself and to acquire, and store on-board, single-lead ECG strips. We have reprogrammed an HR monitor intended for sports use (Movensense HR+) to run our proprietary RITMIA algorithm code in real-time, based on RR analysis, so that if any type of arrhythmia is detected, it triggers a brief retrospective recording of a single-lead ECG, providing tracings of the specific arrhythmia for later consultation. We report the initial data on the behavior, feasibility, and high diagnostic accuracy of this ultra-low weight customized device for standalone automatic arrhythmia detection and ECG recording, when several types of arrhythmias were simulated under different baseline conditions. Conclusions: The customized device was capable of detecting all types of simulated arrhythmias and correctly triggered a visually interpretable ECG tracing. Future human studies are needed to address real-life accuracy of this device.

## 1. Introduction

Technological advances in the field of cardiac rhythm monitoring have been rapid in the last years. The design of new devices or repurposed consumer technology has been focused mostly on the detection of silent atrial fibrillation (AF) [1], but recent studies demonstrate limited clinical utility in the pursuit of short and asymptomatic AF episodes [2]. Still, establishing the diagnosis of several other types of brady- or tachy-arrhythmias remains extremely useful to patients and their caring cardiologists since symptoms such as palpitations or syncope remain among the top reasons for cardiology visits [3,4,5,6]. Most arrhythmias are short-lived, but the prognostic value of detecting even a few seconds of some of them, for example, asystole or ventricular tachycardia, is very high. The usefulness of opportunistic screening strategies, using an electrocardiogram (ECG) or other methods for random “snapshot” assessments, is limited by the unexpected and occasional nature of arrhythmias, leading to a high rate of missed diagnosis [7,8,9,10].

Studies have demonstrated that prolonging the monitoring period yields incremental detection of arrhythmias [11,12], but Holter ECG recordings remain limited in the maximal duration of continuous monitoring, often not sufficient to detect rarely occurring arrhythmias [13].

We have previously validated a cardiac monitoring system (for AF detection) pairing simple consumer-grade Bluetooth low-energy (BLE) heart rate (HR) sensors with a smartphone application (RITMIA™, Heart Sentinel srl, Italy) [14,15]. The application, running on a smartphone, receives beat-to-beat RR interval data by the HR sensor and applies the algorithm in real-time, generating a continuous output of either probable AF, non-AF arrhythmia or normal sinus rhythm, using a combination of RR intervals variability and chaoticity [16,17,18]. Sudden RR interval variation either at inception or during or at the end of the episode is a common feature in most arrhythmias.

In the current study, we aim to test a significant upgrade to the above-mentioned system, thanks to the technical capability of new HR sensors to run algorithms on the sensor itself and to acquire, and store on-board, single-lead ECG strips when asked to do so. Using an existing software development kit (SDK) [19], we have reprogrammed a HR monitor intended for sports use (Movensense HR+) to run our proprietary RITMIA algorithm code in real-time, based on RR analysis, so that any time any type of arrhythmia is detected, it triggers a brief retrospective recording of a single-lead ECG. This provides tracings of the specific arrhythmia for later consultation.

We report the diagnostic accuracy of this affordable, ultra-low weight customized device for standalone automatic arrhythmia detection and ECG recording when several types of arrhythmias were simulated. It was also tested in a healthy adult volunteer exhibiting occasional premature beats.

## 2. Materials and Methods

### 2.1. The Custom Programmed Sensor

The core component of this new diagnostic system is a commercially available Bluetooth low-energy (BLE) HR monitor sensor (Movesense HR+, Suunto, Finland), commercialized for use during sports, working through a chest-strap, although it can also be connected to the skin with small adhesive patch-type electrodes. The sensor can reliably and continuously acquire peak RR cardiac interval data [13] through the electrodes in contact with the skin. The integrated circuits filter out noise and non-cardiac electrical potentials based on frequency or amplitude, robustly selecting and transmitting only R-R interval data in real-time through a BLE standard protocol. Importantly, this device is also able to acquire, transmit and store single-lead electrocardiograms, when prompted to do so.

We programmed this device using the SDK optionally provided with the sensor, embedding our patented algorithm for arrhythmia detection (RITMIA™, Heart Sentinel srl, Parma, Italy) so that only the robust RR interval data, in milliseconds, are used for continuous computation of variability indexes. This real-time rhythm classification of “normal sinus rhythm”, “probable AF” or “undetermined non-AF arrhythmia”, this last category encompassing all events not recognized as either non-normal sinus rhythm or AF. The algorithm uses a moving data matrix comprising a number of prior R-R intervals. It measures RR heterogeneity based on a combination of variability and chaoticity [15]. The device was programmed to acquire 5 s of retrospective ECG strips if the RITMIA™ algorithm recognizes an episode of undetermined non-AF arrhythmia and 10 s retrospective strips at the inception and at the end of an AF episode. When the rhythm is labeled as normal sinus rhythm, no ECG is recorded since no arrhythmia is present, preserving the limited memory of the device.

### 2.2. The Simulation Environment

**Figure 1:**
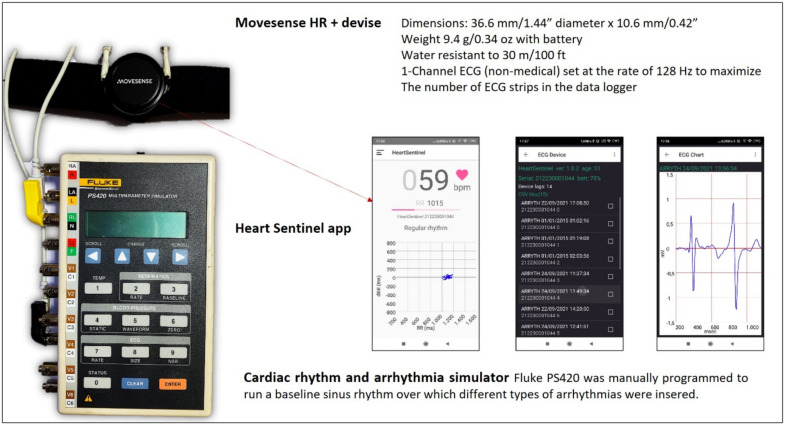
The simulation system components, the Movesense HR+ sensor attached to the chest-belt, and in parallel, wired to the output of a Fluke PS420 simulator. In the middle, three screenshots from the Heart Sentinel app are shown, from left to right, demonstrating: A real-time RR plot, a log of the ECG strips recorded, and a sample of 5-s ECG strip triggered by a premature ventricular beat, obtained after clicking on one of the events in the log. The Heart Sentinel app (in this case running on an Android smartphone) was used during simulations for continuous upload of RR intervals to the cloud, to be later shown in the back-office cloud app matched with stored ECG tracings.

**Figure 2:**
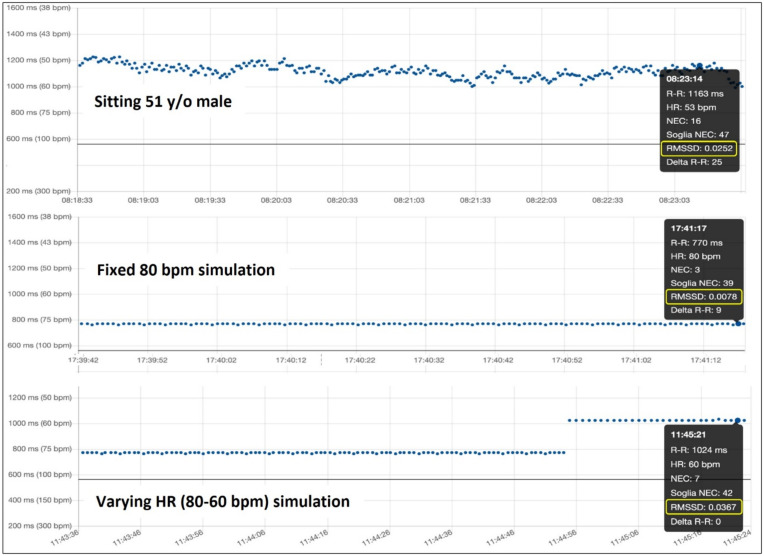
While the physiological heart rate variability extrinsicates in an RMSSD index around 0.025 in an adult male in the sitting position in «real-life» (**top**), the simulation with a fixed heart rate (**middle**) does not account for physiological RR variability (RMSSD 0.0078), while varying heart rate between 80 bpm and 60 bpm in the simulation brings the RMSSD index back to the order of magnitude of physiological values (RMSSD 0.0367) (**bottom**).

Figure 1 shows the simulation environment used. In healthy human subjects, at least some heart rate variability is present, so that, for example, the root mean square of the successive differences among multiple beats (RMSSD) is always higher than zero, typically around 0.025 in a healthy sitting adult. The value measured in our voluntary tester is reported in Figure 2, top. In contrast, the use of an electronically simulated baseline sinus rhythm at a fixed rate shows non-physiological absence of variability (Figure 2, middle). We conducted the arrhythmia simulation tests both using this non physiological fixed-rate sinus rhythm and varying the rate of simulated normal sinus rhythm (Figure 2 bottom), something that simulates the RMSSD with the same order of magnitude of variability as a human sitting adult.

The interrogation of the device to download the ECG strips that the device autonomously acquired was conducted at the end of the arrhythmia simulations, using a BLE-connected Android application (Heart sentinel application). The last ECG recording from the log was discarded because a false alarm is always triggered by artifacts when the device is removed from the body before it goes into standby mode. The RR intervals and their classification as either normal rhythm or one of the two above-mentioned types of arrhythmias (AF or non/AF), based on the RR-based algorithm, were also available from the back-office app on the cloud, where the Heart sentinel app uploads all RR data, if kept connected to the sensor via BLE. In this experimental setting, the sensor both sends the RR intervals to the smartphone app (being uploaded to the back-office cloud app in near real-time) and at the same time, works as a standalone device computing the RR algorithm and triggering the recording of ECGs if an arrhythmia is detected.

## 3. Results

**Figure 3:**
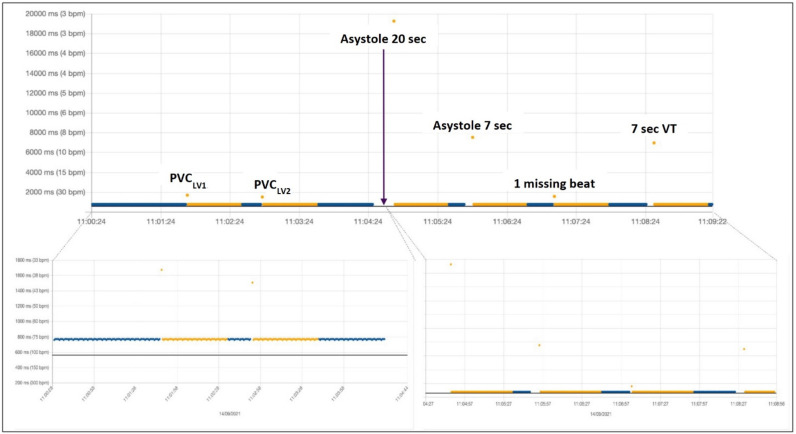
PVC, premature ventricular complex; VT, ventricular tachycardia. Beats considered from normal sinus rhythm are automatically labeled in blue, while non-AF arrhythmia in orange. If AF is present, it is labeled in red. After a single PVC or any other abnormal beat (or pause) recognized as an arrhythmic event by the algorithm, a given number of subsequent beats remain labeled in orange until the last arrhythmic beat exits the moving matrix made by a fixed number of consecutive prior beats, used for computation of variability in real-time. Labeled beats always follow the arrhythmic event, but they can trigger the acquisition of other electrocardiograms only once the rhythm reverts to normal (at least one blue beat).

Figure 3 shows the RR interval results of the sequential simulation of two different types of premature ventricular beats (PVCs), both of left ventricular origin type, a long asystole, lasting 20 s, then another shorter asystole of only 7 s, then a single missing beat and a short (non-sustained) ventricular tachycardia. After each simulated arrhythmic event, the rhythm was reverted to at least several seconds of normal sinus rhythm at 80 beats per minute. The arrhythmia was simulated only after the rhythm was once again recognized as normal (blue labeled).

**Figure 4:**
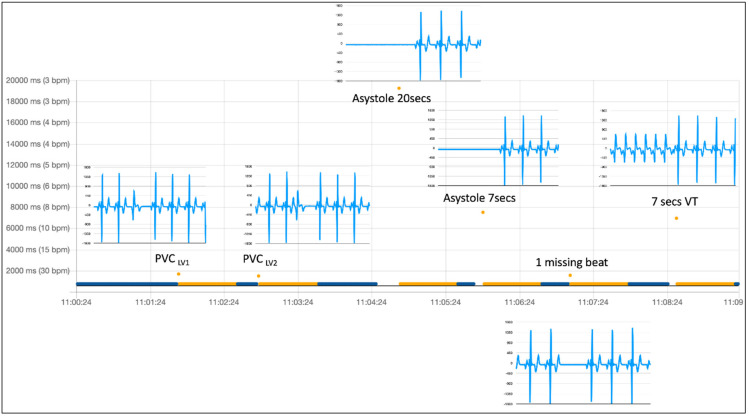
PVC, premature ventricular complex. Beats considered from normal sinus rhythm are automatically labeled in blue, while non-AF arrhythmia in orange. After a single PVC or any other abnormal beat (or pause) recognized as an arrhythmic event by the algorithm, a given number of subsequent beats remain labeled in orange until the last arrhythmic beat exits the moving matrix made by a fixed number of consecutive prior beats, used for computation of variability in real-time. Labeled beats always follow the arrhythmic event, but they can trigger the acquisition of other electrocardiograms only once the rhythm re-verts to normal (at least one blue beat). The missing beat is also recognized.

Figure 4 shows the ECG tracings (5 s each) downloaded from the device at the end of the simulation sequence shown in Figure 3. Tracings are here superimposed on the uploaded RR intervals, according to their timestamp, to match the RR abnormality that triggered retrospective ECG acquisition on the device. All simulated abnormalities effectively triggered an ECG recording, which is clearly diagnostic for the type of simulated arrhythmia. The simulation sequence was repeated three times, and, as expected, the behavior of the device did not vary.

**Figure 5:**
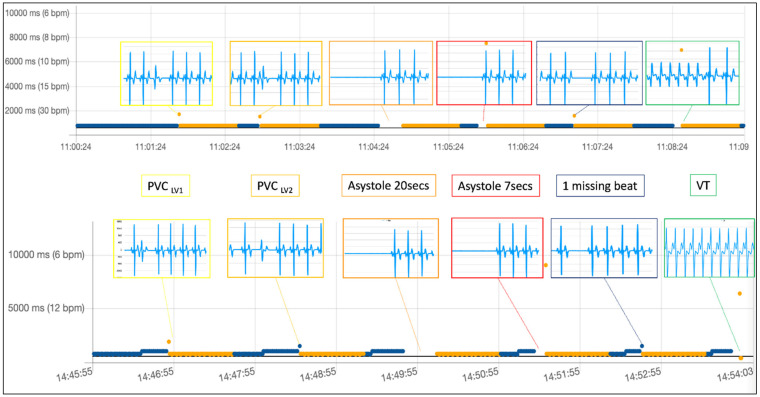
PVC, premature ventricular complex. Beats considered from normal sinus rhythm are automatically labeled in blue, while non-AF arrhythmia in orange. After a single PVC or any other abnormal beat (or pause) recognized as an arrhythmic event by the algorithm, a given number of subsequent beats remain labeled in orange until the last arrhythmic beat exits the moving matrix made by a fixed number of consecutive prior beats, used for computation of variability in real-time. Labeled beats always follow the arrhythmic event, but they can trigger the acquisition of other electrocardiograms only once the rhythm re-verts to normal (at least one blue beat). The missing beat is also recognized.

In Figure 5, we show the same arrhythmia simulation sequence already shown in Figure 3 and Figure 4 but repeated under different baseline conditions. In this case, we simulated a more physiological baseline RR interval behavior during sinus rhythm preceding arrhythmia inception by changing the heart rate of the sinus rhythm from 80 bpm to 60 bpm (as explained in Figure 2) before inserting each of the arrhythmic events in the simulation. One of the parameters used in the RITMIA™ diagnostic algorithm, a measure of heart rate is in fact influenced by RR variability data in the matrix of intervals preceding the event and the conduction of the simulation using the same RR interval, as typical of electronic simulations, is not what happens in real life, where significant beat to beat RR interval variation is present.

Figure 5 top shows the simulation without variating the heart rate (and hence the same RR intervals in the matrix preceding the arrhythmia) while at the bottom, the same sequence of arrhythmic events was simulated, but in this case, only after modifying the baseline heart rate from 80 bpm to 60 bpm in the seconds preceding each simulated arrhythmia.

The results are similarly diagnostic. All events were detected by the algorithm and the acquisition of an ECG strip was always automatically triggered, always including the main arrhythmia event in the limited 5-s duration of the ECG recordings. There are also differences, since for example, “PVC LV2» and «Missing beat» were not immediately recognized as arrhythmias (see the blue color labeling of the PVC beat, meaning no arrhythmia has yet been detected), but the orange labeling arrives in the immediately following beat, a different behavior compared with the fixed-rate artificial sinus rhythm at 80 bpm used in the first set of simulations reported in the top graph. This happens because using a varying heart rate as a baseline sinus rhythm, the different RR of the isolated single arrhythmic event is not sufficiently different to cause the index of RR variability cross the abnormality threshold, requiring a second interval different from the prior mean (the post-event longer RR interval) to push the variability index further towards abnormality, labelling this second beat as “orange” (non-AF arrhythmia) and triggering a retrospective ECG acquisition. Anytime even a single-beat arrhythmia is detected, the “orange” labelling is maintained for several seconds even if no new arrhythmia presents because of the time needed to have the abnormal RR being excluded from the real-time matrix of many prior beats used by the algorithm to compute the RR variability.

**Figure 6:**
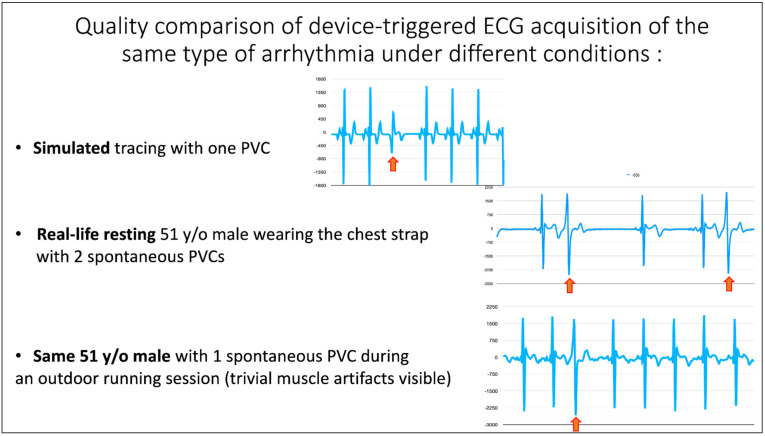
ECG, electrocardiogram; PVC, premature ventricular complex. Quality comparison of device-triggered ECG acquisition of the same type of arrhythmia under different conditions of rest and stress.

Figure 6 shows how the device was able to detect and record arrhythmias during simulations, during real-life with the subject in the sitting position, and, importantly, during sports activity with only trivial deterioration of tracing quality during sports activity.

While noninvasive ultra-light devices are becoming increasingly useful for cardiac monitoring, they share the key limitation of limited built-in memory, so that continuous ECG recording for days or weeks is not possible, at least using the current technology and maintaining the device ultra-portable and light, which is key for patient compliance.

The way we solved this problem, without sacrificing the possibility to have ECG strips available for later cardiology consultation, is by the continuous use of our robust RR-interval algorithm to screen for the presence of a potential arrhythmia, triggering a snapshot recording of short ECG strips only in case arrhythmia is detected, relying on the possibility to acquire retrospective ECG recordings from the data buffer. This makes the memory sufficient for 200–250 ECG strips, which are clinically appropriate for a device meant to screen for infrequent arrhythmias. This mechanism also fully relies on an algorithm based on RR-variability, which is certainly very robust (few false positives) but may be relatively insensitive to those very rare types of arrhythmias not showing RR interval variation at their inception, during the arrhythmia or at the end.

This may be the case, for example, of tachycardias starting at a very similar rate compared with the preceding baseline sinus tachycardia, and not showing at least one sufficiently different RR interval at inception or the end of the arrhythmia, but this is probably clinically rare.

New, noninvasive technologies for cardiac monitoring are flourishing. New devices, such as modified blood pressure monitors, dedicated wearable photoplethysmography-based (PPG) heart rate monitors [20], portable devices for single-lead ECG, traditional but smaller cabled Holter-ECG devices may all find a role in the detection of arrhythmias [21,22,23,24].

However, most of such devices have key limitations, in terms of easy applicability and patient compliance, for screening long-term for arrhythmia detection. Firstly, all of them require specifically dedicated and costly hardware. Secondly, most devices (with the exclusion of continuous Holter-ECG monitors) need active participation of the patient to trigger recording, reducing their utility for the detection of asymptomatic or unexpected and occasional arrhythmias. Thirdly, if they record continuously, as Holter monitors do, the period they can monitor and record is limited in duration, again with less-than-optimal capability to discover occasional paroxysmal events, which may take place as rarely as a few times in a year [25].

Rare but clinically dangerous syncopal or pre-syncopal episodes, for example, are almost impossible to be recorded with external Holter monitors [26], although detecting asystole may signal the need for a pacemaker placement. An ultralight and standalone device like the one we prototyped may be applied at the patient’s convenience anytime he prefers, even for months, with the simple caution to change the very inexpensive battery once in a while (once a month or less) and the comfortable adhesive patch, if used because the chest strap is less comfortable.

## 4. Conclusions and Future Work

The present feasibility and initial validation study tested a new standalone device, in its original iteration commercially available as a HR chest-belt sensor for sports [18]. We modified its software embedding our patented arrhythmia-detection algorithms, which makes this potentially the smallest and lightest device capable of detecting arrhythmias and record them in the single-lead ECG tracing format [27].

The device is reusable, shockproof, and waterproof, and the coin-sized battery easily lasts more than a month and can be easily substituted by the user (without loss of on-board recordings).

Future work should validate the effectiveness of this device on human subjects and in real-life condition.

## Data Availability

Data access is restricted due intellectual property concerns. Data can be obtained after motivated request.
